# Benfotiamine Attenuates Inflammatory Response in LPS Stimulated BV-2 Microglia

**DOI:** 10.1371/journal.pone.0118372

**Published:** 2015-02-19

**Authors:** Iva Bozic, Danijela Savic, Danijela Laketa, Ivana Bjelobaba, Ivan Milenkovic, Sanja Pekovic, Nadezda Nedeljkovic, Irena Lavrnja

**Affiliations:** 1 Department of Neurobiology, Institute for Biological Research “Sinisa Stankovic”, University of Belgrade, Belgrade, Serbia; 2 Institute for Physiology and Biochemistry, Faculty of Biology, University of Belgrade, Belgrade, Serbia; 3 Carl Ludwig Institute for Physiology, Faculty of Medicine, University of Leipzig, Leipzig, Germany; National Brain Research Center, INDIA

## Abstract

Microglial cells are resident immune cells of the central nervous system (CNS), recognized as key elements in the regulation of neural homeostasis and the response to injury and repair. As excessive activation of microglia may lead to neurodegeneration, therapeutic strategies targeting its inhibition were shown to improve treatment of most neurodegenerative diseases. Benfotiamine is a synthetic vitamin B1 (thiamine) derivate exerting potentially anti-inflammatory effects. Despite the encouraging results regarding benfotiamine potential to alleviate diabetic microangiopathy, neuropathy and other oxidative stress-induced pathological conditions, its activities and cellular mechanisms during microglial activation have yet to be elucidated. In the present study, the anti-inflammatory effects of benfotiamine were investigated in lipopolysaccharide (LPS)-stimulated murine BV-2 microglia. We determined that benfotiamine remodels activated microglia to acquire the shape that is characteristic of non-stimulated BV-2 cells. In addition, benfotiamine significantly decreased production of pro-inflammatory mediators such as inducible form of nitric oxide synthase (iNOS) and NO; cyclooxygenase-2 (COX-2), heat-shock protein 70 (Hsp70), tumor necrosis factor alpha α (TNF-α), interleukin-6 (IL-6), whereas it increased anti-inflammatory interleukin-10 (IL-10) production in LPS stimulated BV-2 microglia. Moreover, benfotiamine suppressed the phosphorylation of extracellular signal-regulated kinases 1/2 (ERK1/2), c-Jun N-terminal kinases (JNK) and protein kinase B Akt/PKB. Treatment with specific inhibitors revealed that benfotiamine-mediated suppression of NO production was via JNK1/2 and Akt pathway, while the cytokine suppression includes ERK1/2, JNK1/2 and Akt pathways. Finally, the potentially protective effect is mediated by the suppression of translocation of nuclear factor kappa-light-chain-enhancer of activated B cells (NF-κB) in the nucleus. Therefore, benfotiamine may have therapeutic potential for neurodegenerative diseases by inhibiting inflammatory mediators and enhancing anti-inflammatory factor production in activated microglia.

## Introduction

Microglia are resident immune cells in the central nervous system (CNS), involved in its immune surveillance and continuous scanning for signs of danger [1,2]. These cells play a pivotal role in the CNS innate immunity and serve as the first line of defense against invading pathogens [3]. In a pathological context, activation of microglia involves proliferation, migration to the site of injury, increased expression of immunomodulators and transformation into phagocytes capable of clearing damaged cells and debris [4]. However, excessive inflammation involving microglia activation may lead to a vicious cycle of neuroinflammation that contributes to neurodegeneration [1]. Upon activation, microglia also undergoes dramatic morphologic changes, from resting ramified shape into activated amoeboid morphology [5,6,7]. These changes are concomitant with up-regulation of several transcription factors (e.g. NF-κB) and release of soluble factors, such as proinflammatory cytokines, chemokines [8] and reactive oxygen species [9]. Together, these processes play a critical role in the neuronal damage in various neurodegenerative diseases [10]. Therefore, the activation of counter-regulatory mechanisms is essential in preventing escalation of inflammatory processes [11], thus pointing to the importance of scrutinizing the molecular mechanisms underlying the microglia activation and de-activation. Consequently, it is important to investigate the negative regulators of microglial activation and their underlying molecular mechanisms.

The significance of vitamin B1 (thiamine) in glucose metabolism, neurotransmission and neurological function in CNS is well known [12,13]. The largest amount of this vitamin in CNS is found in cell membrane, where it has a role in regeneration of damaged cells [12]. Although all cell types utilize thiamine, the nervous system is particularly sensitive to thiamine deficiency pertaining to impaired oxidative metabolism, altered neuron function, blood–brain barrier disruption, astrocyte dysfunction, excitotoxicity, amyloid deposition and inflammation [14,15]. Thiamine deficiency is associated with Wernicke-Korsakoff syndrome, Alzheimer’s disease, amyotrophic lateral sclerosis, Parkinson’s disease, multiple sclerosis and diabetes [16,17], which are treated with effectiveness with thiamine or its derivatives. Furthermore, the relationship between thiamine deficiency and microglial activation has been established in animal studies [18,19]. Benfotiamine (*S*-benzoylthiamine O-monophosphate) is a synthetic S-acyl derivative of vitamin B1 with a much higher bioavailability than genuine thiamine [20,21,22,23,24]. Due to the open thiazole ring, benfotiamine has a high lipid solubility enabling it to reach, to a much higher degree than the water-soluble salts, several organs in animals and humans [25,26]. Originally, benfotiamine was developed in Japan to treat alcoholic neuropathy and other painful neurological complications [27]. Nowadays benfotiamine is largely used for treatment of diabetic neuropathy, nephropathy, retinopathy and cardiac angiopathy [23,28,29]. During the last few years, there was considerable interest in the therapeutic potential of benfotiamine and its protective effect was elucidated in diabetic complications, such as diabetic neuropathy [29] and alcoholic neuropathy [30]. Also, its beneficial effect was shown in the animal model of Alzheimer’s disease. Benfotiamine significantly reduced the formation of amyloid plaques in APP/PS1 mice [31] and was able to attenuate the glucose-induced increase in β-amyloid protein synthesis in isolated HEK293 cells [32].

Although the protective role of benfotiamine has been documented, the involvement of benfotiamine potential effects on activated microglia remained elusive. Given the anti-inflammatory and anti-oxidative potency of benfotiamine [33,34,35], we determined in the present study the molecular mechanism underlying its protective role in LPS activated BV-2 microglia. Our data demonstrate that benfotiamine inhibited microglial activation through attenuated production of NO and expression of iNOS, Cox-2, Hsp 70 and decreased expression and release of TNF-α and IL-6 by blocking ERK1/2, JNK and Akt/PKB signaling pathway and NF-κB activation induced by LPS in BV-2 cells. Our results indicate a potential role of benfotiamine in neuroprotection via its anti-neuroinflammatory effect. This hypothesis needs to be validated in an in vivo model in future studies.

## Materials and Methods

BV-2 microglial cell line was developed by immortalizing primary mouse microglial cells with v-raf/v-myc recombinant retrovirus, in the laboratory of Dr Blasi [36] and was a generous gift from Dr Alba Minelli (University of Perugia, Perugia, Italy). Cells were maintained in RPMI 1640 medium (GE Healthcare Life Sciences, Freiburg, Germany) supplemented with 10% heat-inactivated fetal bovine serum (FBS, PAA Laboratories GmbH, Pasching, Austria) and 1% penicillin/streptomycin (Invitrogen, Carlsbad, CA, USA) at 37°C in a humidified incubator under a 95% air/5% CO_2_. When cells reached approximately 80% confluence, they were detached with 0.1% trypsin-EDTA (PAA Laboratories GmbH, Pasching, Austria), seeded into appropriate dishes and incubated overnight. Then BV-2 cells were pre-treated for 30 min with different concentrations of benfotiamine (Sigma-Aldrich, Munich, Germany; 50, 100 or 250 μM) before stimulation with LPS from *Escherichia coli* serotype 026:B6 (Sigma-Aldrich, Munich, Germany; 1μg/ml). Incubation time with LPS varied depending on the purpose of the experiment.

### Cell viability and cell morphology

Cell viability and morphology was evaluated using xCELLigence Real-Time Cell Analyzer Single Plate instrument (RTCA SP, ACEA Biosciences, San Diego, CA, USA). This system enables analysis of the cell status in real-time by impedance measurement through gold microelectrodes on the bottom of each well of an E-plate 96 (ACEA Biosciences, San Diego, CA, USA). The interaction of cells with microelectrodes generates a impedance that is expressed as a Cell Index value correlating with the number, viability, morphology and adhesion of the cells. Cells were seeded at 1 x 10^4^ per well, incubated overnight and thereafter pretreated with benfotiamine for 30 min prior to stimulation with LPS for 24 hours. Cell Index was recorded every 5 min during the whole experiment. The same medium without a cell culture served as the background. Results were expressed as Normalized Cell Index calculated as the Cell Index at a given time point divided by the Cell Index at the time point of LPS administration.

In order to examine whether the differences in Cell Index values between the groups measured after 24 h of LPS stimulation were caused by the changes in cell viability, we performed crystal violet assay. BV-2 cells were seeded in 96 well plates (1 x 10^4^ cells/well), pre-treated with benfotiamine and stimulated with LPS for 24h. Cells were briefly washed with PBS and then fixed with 4% paraformaldehyde for 20 min, at 4°C. Subsequently, cells were stained with 1% crystal violet solution (Sigma-Aldrich, Munich, Germany) for 15 min, washed with water and then dried overnight. The next day, the dye bound to the cells was dissolved with 33% acetic acid and absorbance was measured at 540 nm with the reference wavelength at 640 nm, using a microplate reader (LKB 5060–006, Vienna, Austria).

At the same time point, cell morphology was analyzed with phase contrast and fluorescence imaging of cytoskeleton. BV-2 cells were plated at 8 x 10^4^ on glass cover-slips (Ø25 mm) in 35 mm dishes (Sarstedt, Newton, NC, USA). After 24h treatment cells were washed with PBS and phase contrast images were immediately acquired. For immunofluorescence cells were fixed with 4% paraformaldehyde for 20 min at 4°C, washed with PBS and then permeabilized with Triton X-100 (0.25%, Sigma-Aldrich, Munich, Germany) for 15 min. Filamentous F-actin was stained with Alexa Fluor 555 phalloidin (Invitrogen, Carlsbad, CA, USA, 1:50 dilution in PBS, for 30 min). After washing with PBS, nuclear counterstain with Hoechst 33342 (5 μg/ml, Life Technologies, Invitrogen, Carlsbad, CA, USA) was performed. Cells were cover-slipped with Mowiol (Calbiochem, Darmstadt, Germany) and images were acquired using Zeiss Axiovert fluorescent microscope (Zeiss, Jena, Germany).

Images of cells stained with phalloidin were used to quantify the average cell surface in each group, using the AxioVisionRel 4.6 software (Zeiss, Jena, Germany). Cells were analyzed in five areas (138 x 104 μm^2^) per cover-slip, with three cover-slips for each group, in three independent sets of experiments.

### Immunofluorescent labeling and quantification of fluorescence intensity

For immunofluorescence, cells were pre-treated with various concentrations of benfotiamine and stimulated with LPS for 30 min (for detection of NF-κB/p65 translocation) and 24h (for detection of iNOS). Afterwards, cells were fixed, washed, permeabilized as stated previously and blocked with 5% bovine serum albumin (BSA, Sigma-Aldrich, Munich, Germany). Primary antibodies against NF-κB/p65 or iNOS were applied overnight at 4°C (dilutions and specifications are given in Table 1). The next day cells were incubated with appropriate fluorophore—labeled secondary antibody (Table 1) for 1 h at room temperature. Cells were rinsed with PBS; nuclei were counterstained with Hoechst 33342 and after washing cover-slips were mounted with Mowiol. Negative controls underwent the same procedure without incubation with primary antibodies.

**Table 1:** List of primary and secondary antibodies used for immunofluorescence (IF) and western blot (WB).

Antigen	Source	Dilution	Company
iNOS	rabbit	1:100 IF; 1:500 WB	abcam, ab15323
NFKB/p65	rabbit	1:200 IF; 1:2000 WB	Santa Cruz, sc-372
phospho-p44/42 MAPK	rabbit	1:2000 WB	cell signalling, 4370
p44/42 MAPK	rabbit	1:1000 WB	cell signalling, 4695
phospho-SAPK/JNK	rabbit	1:1000 WB	cell signalling, 4668
SAPK/JNK	rabbit	1:1000 WB	cell signalling, 9258
phospho-p38	rabbit	1:1000 WB	cell signalling, 9215
p38	rabbit	1:1000 WB	cell signalling, 9212
phospho-Akt	rabbit	1:1000 WB	cell signalling, 9275
Akt	rabbit	1:1000 WB	cell signalling, 9272
Cox-2	rabbit	1:1000 WB	Santa Cruz, sc-7951
Hsp 70	goat	1:1000 WB	Santa Cruz, sc-1060
lamin B	goat	1:1000 WB	Santa Cruz, sc-6217
β-tubulin	goat	1:2000 WB	Santa Cruz, sc-9935
β-actin	mouse	1:5000 WB	Sigma-Aldrich, A2228
anti-goat IgG-HRP	donkey	1:5000 WB	Santa Cruz, sc-2020
anti-rabbit IgG-HRP	donkey	1:5000 WB	Santa Cruz, sc-2305

NF-κB/p65 fluorescence intensity in the nucleus was quantified with Image J software as previously described [37]. Fluorescence intensity of nuclear NF-κB/p65 was measured in at least 200 hundred cells per each experimental group and the results were presented in arbitrary units. The data were binned (5 AU steps) according to fluorescence intensity and represented as cumulative percentage.

### Measurement of nitric oxide production

Production of NO was determined by measuring nitrite levels as a stable NO product, using the Griess reagent (1% sulphanilamide, Sigma-Aldrich, Munich, Germany, and 0.1% N-(naphthyl)-ethylenediaminedihydrochloride, Fluka, Buchs, Switzerland in 2% H_3_PO_4_). BV-2 microglial cells were seeded in 24-well plates (5 x 10^4^ cells/well) and treated with benfotiamine for 30 minutes before application of LPS for 24 hours. Then, the cell culture medium was collected and mixed in equal volume with Griess reagent. Following 10 min incubation in the dark the absorbance at 570 nm was measured. Increasing concentrations of sodium nitrite were used to generate a standard curve from which the nitrite concentration in the medium was calculated.

### Quantitative real-time PCR

BV-2 cells were seeded in 6-well plates at a density of 3 x 10^5^ cells/well, treated with benfotiamine and/or LPS and harvested after 6 hours. Total RNA was extracted with TRIzol reagent (Invitrogen, Carlsbad, CA, USA) according to the manufacturer’s protocol. Concentration of RNA was determined by measuring absorbance at 260 nm and 1 μg of RNA was used for cDNA synthesis (High Capacity cDNA Reverse Transcription Kit, Applied Biosystems, Foster City, CA, USA). Real-time PCR amplifications were performed in triplicate, using a mixture of SYBR Green PCR Master Mix (Applied Biosystems, Foster City, CA, USA), cDNA samples and designate primers (sequences given in Table 2, Invitrogen, Carlsbad, CA, USA). Reactions were conducted in the ABI Prism 7000 Sequence Detection System (Applied Biosystems, Foster City, CA, USA). Relative gene expression was calculated by comparing C_T_ value of the gene of interest to the C_T_ value of GAPDH, internal control (the 2^-ΔΔCT^ method). The PCR products were run on 2% agarose gels and visualized under the UV light (data not shown).

**Table 2:** List of primers used for Real Time-PCR.

Gene	Forward primer	Reverse primer	bp	Annealing T(°C)
TNF-α	GCCCACGTCGTAGCAAACCAC	GGCTGGCACCACTAGTTGGTTGT	117	64
IL-6	TAGTCCTTCCTACCCCAATTTCC	TTGGTCCTTAGCCACTCCTTC	76	60
IL-10	GCTCTTACTGACTGGCATGAG	CGCAGCTCTAGGAGCATGTG	105	60
iNOS	GGTGTTCTTTGCTTCCATGCTAAT	GTCCCTGGCTAGTGCTTCAGA	106	60
PTGS2	TTCAACACACTCTATCACTGGC	AGAAGCGTTTGCGGTACTCAT	271	64
GAPDH	GTTGTCTCCTGCGACTTCA	TGGTCCAGGGTTTCTTACTC	182	60

### Enzyme-linked immunosorbent assay (ELISA)

For assessment of cytokine production BV-2 cells were seeded in 6 well plates (3 x 10^5^ cells/well), pre-treated with benfotiamine, and stimulated with LPS for 24 h. Thereafter, the cell culture medium was collected and concentrations of TNF-α, IL-6 and IL-10 were determined with ELISA. The production of TNF-α was measured using a pair of capture and detection antibodies (eBioscience, Frankfurt, Germany) according to the manufacturer’s protocol. After incubation with biotinylated detection antibody, avidin-HRP conjugate and subsequently chromogenic substrate 3,3′,5,5′-Tetramethylbenzidine (TMB, eBioscience, Frankfurt, Germany) were added. Color formation was stopped with 1M H_3_PO_4_ and absorbance was measured at 450 nm. The concentration of TNF-α in cell culture medium was determined from the standard curve obtained with recombinant murine TNF-α. The production of IL-6 and IL-10 was assessed using Mini ELISA Development Kits (Peprotech, Hamburg, Germany) according to the manufacturer’s protocol. The protocol was the same as for determination of TNF-α, except for using the 2,2′-azino-bis(3-ethylbenzothiazoline-6-sulphonic acid) (ABTS, Sigma-Aldrich, Munich, Germany), as a chromogenic substrate. Accordingly, absorbance was measured at 405 nm with correction set at 650 nm. Appropriate standard curves were constructed with recombinant murine cytokines to estimate concentration in the samples.

### Western blot analysis

BV-2 cells were seeded in 6 well plates (3 x 10^5^ cells/well), pre-treated with benfotiamine and stimulated with LPS for 30 min for detection of NF-κB/p65. For detection of proteins in MAPK signaling pathway, LPS incubation lasted for 5, 15, 30 and 60 min. For detection of COX-2 cells were stimulated for 24h. Cytosolic and nuclear extracts were prepared for detection of p65/NF-κB, using Nuclear and Cytoplasmic Extraction Reagents kit (NE-PER, Thermo Scientific, *Waltham*, *MA*, USA). Proteins in MAPK signaling pathway, as for the COX-2 were detected after lysing the cells with ice-cold lysis Triton X-100 buffer (50 mM Tris–HCl, pH 7.4, 150 mM NaCl, 1% Triton X-100, 0.1% sodium dodecylsulphate (SDS)) containing protease (Roche, Penzberg, Germany) and phosphatase inhibitors (Pierce Biotechnology, Rockford, IL, USA). Cell lysates were centrifuged at 17900*g* for 20 min at 4°C, and supernatants were collected. Protein content was determined using the BCA protein assay kit (Pierce Biotechnology, Rockford, IL, USA). Equal protein amounts (20 μg) were loaded into the wells of 7.5% polyacrylamide gels. Following electrophoresis at 100–120 V, proteins were transferred to a polyvinylidene fluoride (PVDF) membrane (Roche, Penzberg, Germany) for 1 h at 100 V with cooling. The membranes were blocked with 5% BSA dissolved in Tris-buffered saline Tween-20 (TBST) (20mMTris, pH 7.6, 136mMNaCl, 0.1% Tween 20) for 1 h at room temperature and incubated overnight with primary antibodies (Table 1). After washing step with TBST, membranes were incubated with appropriate HRP-conjugated secondary antibodies for 1 h at room temperature. Protein bands were visualized using chemiluminescence and developed onto the film (KODAK, Rochester, NY, USA). The relative expression levels of proteins were determined by densitometry and were normalized by comparing to β-tubulin or β-actin of the same lane. Data presented in graphs are mean values ± standard error of the mean obtained from four independent immunoblots.

### Treatment with inhibitors of ERK1/2, JNK and Akt signaling pathways

BV-2 cells were seeded in 6 well plates (3 x 10^5^ cells/well), treated with specific inhibitors for ERK1/2 (U0126, Cell signaling, Danvers, MA, USA, #9910, final concentration 50 μM), JNK (SP600125, Biaffin GmbH & Co KG, Kassel, Germany, final concentration 20 μM) and Akt (LY294002 Cell signaling, USA, #9901, final concentration 20 μM) for 30 min with subsequent incubation with benfotiamine (250 μM) for 30 minutes and stimulated with LPS. Total RNA was extracted 6h after LPS treatment of the cells. For assessment of NO, TNF-α and IL-6 production cell culture medium was collected after 24h of LPS treatment.

### Data analysis

Except where stated otherwise, results are expressed as mean values ± standard error from three independent experiments each run in triplicate. The statistical significance of the differences was evaluated by analysis of variance followed by Bonferroni’s multiple comparison test. Values of *P*<0.05 were considered to be statistically significant.

## Results

### Benfotiamine alters cell morphology in LPS-stimulated BV-2 cells by inducing reorganization of F-actin cytoskeleton

**Fig 1:**
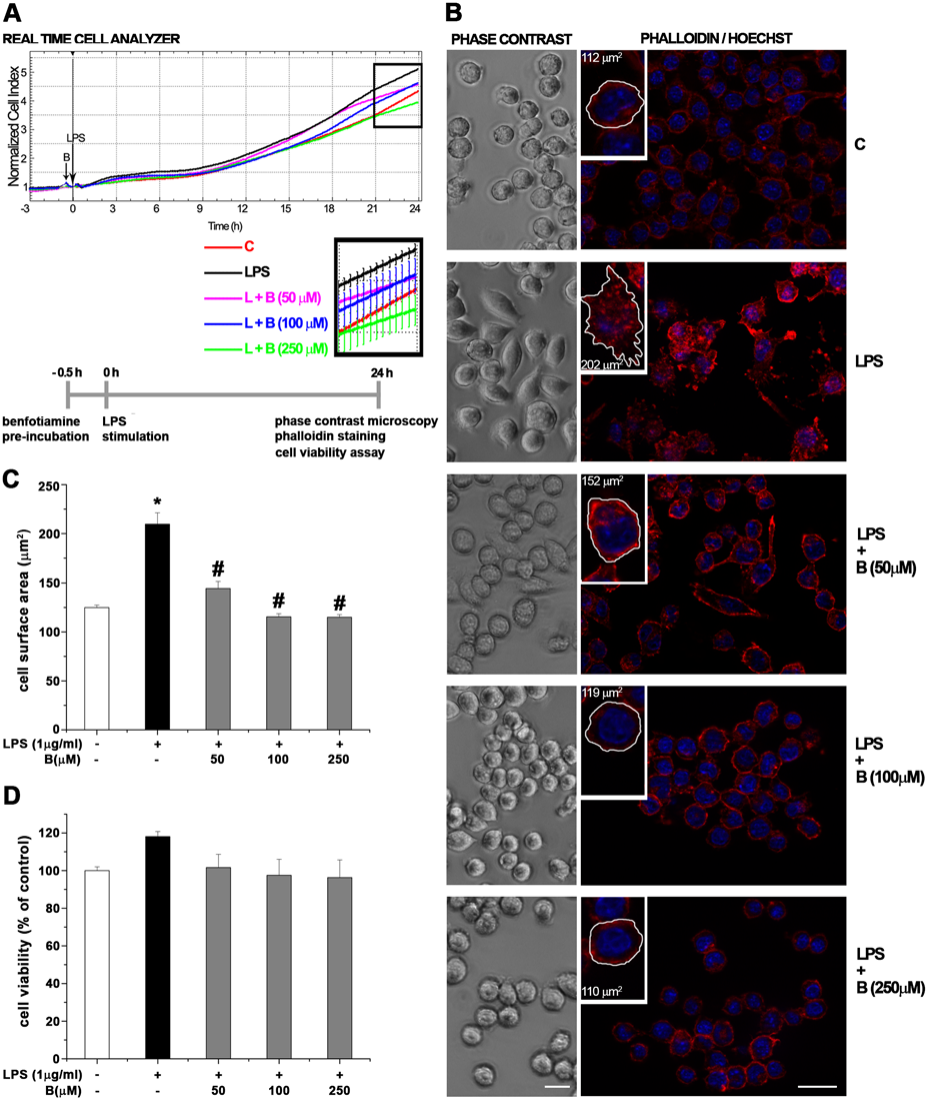
Functional characterization of benfotiamine effects in LPS-stimulated BV-2 microglia. (**A**) Real-time monitoring of BV-2 cell viability using xCELLigence RTCA analyzer. Representative graph showing the rate of proliferation in cells incubated in control medium (red line), medium with 1 μg/ml LPS (black line), or cells pretreated with benfotiamine, 50 μM (pink line), 100 μM (blue line) or 250 μM (green line) and then treated with LPS for 24 h. (**B**) Benfotiamine- induced alterations in cell morphology were analyzed using phase-contrast microscopy (left panels), whereas cell surface area was quantified by Phalloidin /Hoechst fluorescent staining (red/blue) microscopy (right panels), using AxioVisionRel 4.6 software. Insets: cell surface area was measured in five areas (138 × 104 μm^2^) per each cover-slip (n = 3) per experimental group in three independent experiments. (**C**) Bars present mean surface areas (± SEM) obtained from data presented in Fig. 1B. (**D**) Cell viability was assessed by crystal violet staining and results are displayed as percentage of control ± SEM (n = 3). *P < 0.05 control vs. LPS-induced BV-2 cells, # LPS vs. benfotiamine pretreated LPS activated BV-2 cells. Scale bar: 20 μm.

The influence of benfotiamine on cell viability and morphology of control and LPS-treated BV-2 cells was determined using RTCA, which monitors real—time changes in cell impedance (Fig. 1A; S1A Fig.), reflecting the changes in cell number/viability and morphology. The measurements revealed a time-dependent cell index increase, which was most pronounced in LPS-stimulated microglia. Benfotiamine in the absence of LPS revealed no significant changes in cell index of BV-2 cells (S1A Fig.). Pretreatment with benfotiamine alleviated the LPS-induced cell index increase in all dosages, with 250 μM benfotiamine inducing the cell state comparable to one in control culture. Since alteration in cell index reflects either significant morphological changes or decrease in cell viability, phalloidin/Hoechst 33342 double fluorescent staining of F-actin and the viability assay were performed. BV-2 microglial cells display amoeboid, round morphology with uniform, punctuated distribution of F-actin in control cells. Benfotiamine pretreatment had no influence on cell morphology (S1B Fig.). However, benfotiamine induced striking alterations in cell morphology, from large cells with multiple processes, terminating with prominent microvilli, as evidenced in LPS group (Fig. 1B), to round or amoeboid, smooth-surface cells, evidenced in the control (Fig. 1B). Closer examination revealed that benfotiamine reduced dense fasciation of F-actin fibers underneath plasmalemma and stimulated their discrete relocalization throughout the cytoplasm (Fig. 1B). Since F-actin fibers critically determine cellular morphology, postulated benfotiamine-induced morphological changes can be quantitatively expressed as alternations in the cell surface area (Fig. 1C). Indeed, benfotiamine induced a decrease in mean cell surface area compared to LPS-treated BV-2 cells. Crystal violet and trypan blue exclusion viability assay revealed that cell viability of BV-2 cells exposed with or without LPS was not affected in the presence of benfotiamine, in neither one of the concentrations tested (Fig. 1D; S1D and S2 Figs.). Taken together, these data provide evidence that benfotiamine alleviated LPS-induced morphological changes in LPS-stimulated BV-2 cells by inducing reorganization of F-actin cytoskeleton.

### Benfotiamine decreases LPS-induced production of NO by suppressing iNOS-mRNA and protein level

**Fig 2:**
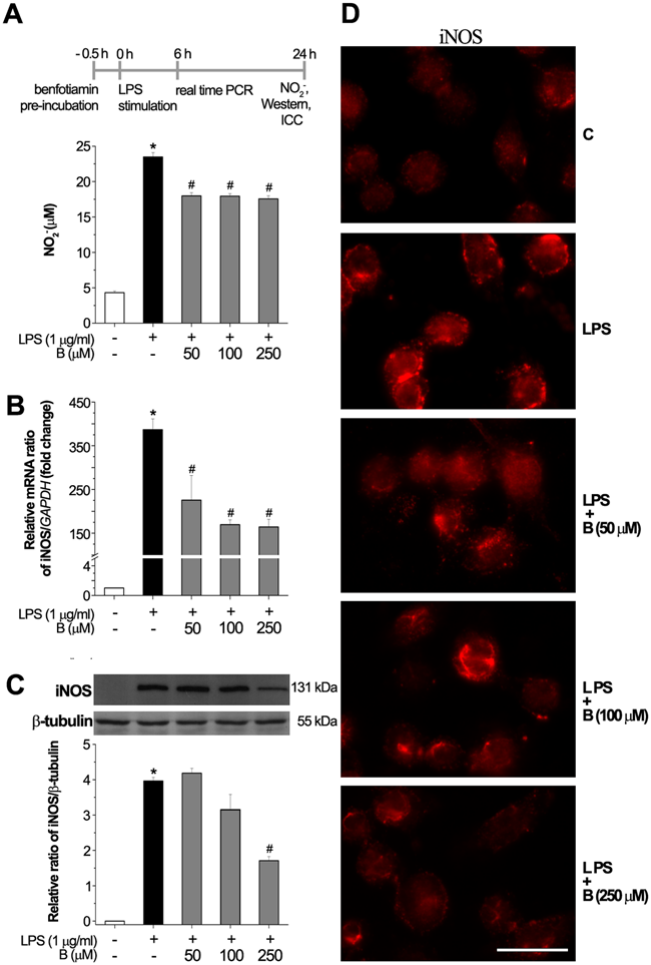
Effect of benfotiamine on LPS-induced production of NO. (**A**) Benfotiamine suppressed LPS-induced release of NO. (**B**) Expression of iNOS-mRNA in LPS-stimulated BV-2 cells (black bar) and cells pretreated with benfotiamine (gray bars). The levels of iNOS-mRNA are expressed relative to the expression of GAPDH-mRNA as an internal control. (**C**) Expression of iNOS at the protein level, as determined by Western blot. Graph shows mean iNOS protein abundance (± SEM), from *n* = 3 separate determinations, expressed relative to the abundance of β-tubulin in each lane. Representative Western blot of iNOS expression. (**D**) Immunofluorescence labeling of BV-2 cells against iNOS. Significance inside the graphs: *p < 0.05 control *vs*. LPS-induced BV-2 cells, # LPS *vs*. benfotiamine pretreated LPS activated BV-2 cells. Scale bar: 20 μm.

To evaluate the effect of benfotiamine on extracellular NO production in BV-2 cells in presence or absence of LPS, the culture medium was collected and concentration of nitrite was determined by the Griess method. BV-2 cells were pre-treated with benfotiamine (50, 100 and 250 μM) for 30 min in presence or absence of LPS (1 μg/ml) for 24 h. Such prolonged treatment with LPS was chosen to allow for changes at the NO level which are determined by the gene- and the protein-expression of iNOS. As shown in S4A Fig., benfotiamine alone did not lead to any change in NO production, whereas LPS significantly induced the generation of NO in BV2 cells. The results indicated that extracellular NO increased in LPS-treated BV-2 cells compared to the control group (P <0.001) (Fig. 2A), whereas, pre-treatment with benfotiamine before exposure to LPS suppressed the production of nitrite (by 25%, P <0.001), irrespective of the concentration of benfotiamine applied.

NO is generated by catalytic action of iNOS, wherein the expression of iNOS is increased by inflammatory factors, such as LPS. To explore whether benfotiamine affects NO production by interfering with expression of iNOS, we determined the mRNA level of iNOS by RT-PCR. Moreover, the protein level of iNOS was assessed by Western blot analysis and immunofluorescent labeling. The cells were pre-treated with benfotiamine (50, 100, 250 μM) for 30 min and then exposed to LPS (1 μg/ml) for 6 h. The expression of *iNOS*-mRNA induced by LPS was significantly reduced (approximately by 42% for 50 μM dose, P<0.05 and 57% for 100 and 250 μM doses, P<0.01) at all applied concentrations of benfotiamine (Fig. 2B). The level of iNOS protein was determined in cells pre-treated with benfotiamine for 30 min and treated with LPS for 24 h. The increased levels of the iNOS protein induced by LPS were reduced by benfotiamine pre-treatment only in the presence of 250 μM benfotiamine (Fig. 2C, 2D). Together, these results are consistent with the hypothesis that benfotiamine down regulates NO production by reducing expression of iNOS.

### Benfotiamine suppresses LPS-induced PTGS mRNA expression and COX-2 protein expression in BV2 microglial cells

**Fig 3:**
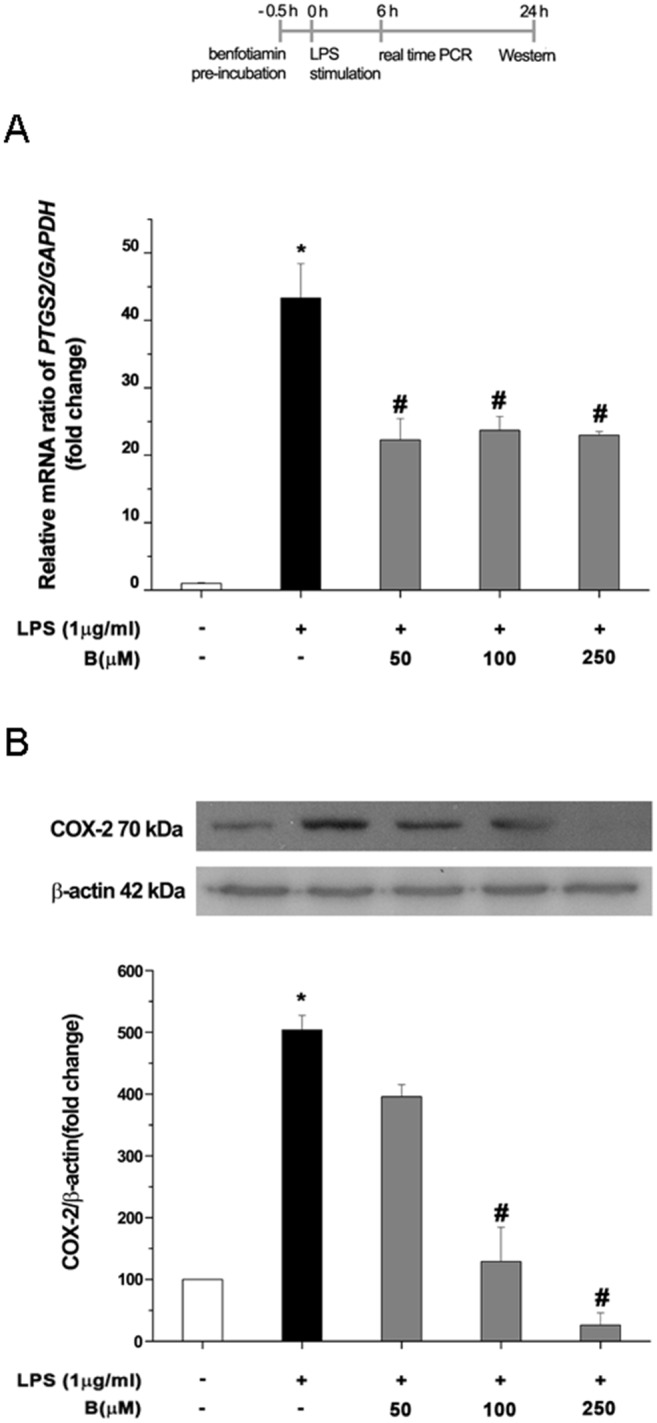
The effect of benfotiamine on LPS—induced expression of proinflammatory effector molecules. (**A**) Expression of prostaglandin—endoperoxidase synthase 2 (PTGS2) at mRNA level in BV-2 cells. Expression of PTGS2-mRNA was assessed by RT-PCR, in control culture (white bar), LPS-treated culture (black bar) and cultures pre-treated with benfotiamine, 6 h following addition of LPS. PTGS2-mRNA abundance was expressed relative to the abundance of GAPDH-mRNA, as an internal control. (**B**) Expression of COX-2 at the protein level, determined by Western blot analysis. Bars show Cox-2/β-actin expression ratio relative to control (100%) ± SEM, from *n* = 3 separate determinations. Significance levels shown inside the graphs: *p < 0.05 control *vs*. LPS-induced BV-2 cells, # LPS *vs*. benfotiamine pretreated LPS activated BV-2 cells.

Since high levels of NO modulate the expression of COX-2, which is another effector molecule implicated in inflammatory neuropathology, we assessed the influence of benfotiamine on LPS induced prostaglandin-endoperoxide synthase 2 (PTGS2) mRNA and COX-2 expression (Fig. 3). The cells were pre-treated with benfotiamine (50, 100, 250 μM) for 30 min and then exposed to LPS (1 μg/ml) for 6 h. The expression levels of the PTGS mRNA were significantly increased following LPS treatment. Benfotiamine substantially reversed the LPS-induced upregulation of PTGS mRNA in all examined dosages by 47% (Fig. 3A). The level of COX-2 and Hsp70 protein was determined in cells pre-treated with benfotiamine for 30 min and incubated with LPS for 24 h. The increased levels of the COX-2 and Hsp70 protein induced by LPS were reduced by benfotiamine in the presence of 100 and/or 250 μM doses, respectively (Fig. 3B; S6A, B Fig.).

### Benfotiamine modulates LPS-induced production and release of cytokines

**Fig 4:**
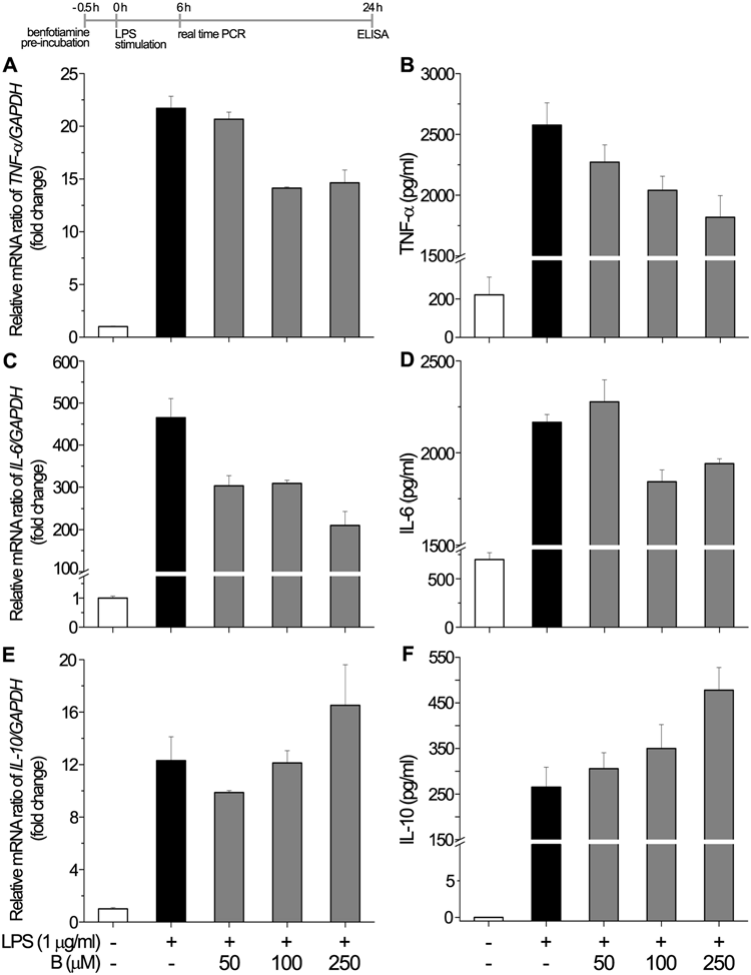
Effect of benfotiamine on cytokines expression and the release by BV-2 cells. Expression of TNF-α (A, B), IL-6 (C, D) and IL-10 (E, F) was analyzed at mRNA (A, C, E) and protein (B, D, F) level. Abundance of each mRNA transcript was expressed relative to GAPDH as internal control. Release of the cytokines was determined in the culture supernatants by ELISA. Bars represent mean ± SEM from *n* = 3 separate determinations. Significance levels shown inside the graphs: * - *p* < 0.05 control *vs*. LPS-induced BV-2 cells; # — LPS *vs*. benfotiamine pretreated LPS activated BV-2 cells.

Production and release of cytokines plays a central role in the microglia-mediated inflammatory action. Hence, the anti-inflammatory potential of benfotiamine was evaluated by assessing its effect on the expression of several master microglia cytokines. The expression of TNF-α, IL-6 and IL-10 was analyzed using quantitative real-time PCR and ELISA. Prior studying the impact of benfotiamine on LPS-induced production of proinflammatory cytokines, we examined its effect on non-stimulated cells in regard to *TNF-α* and *IL-6* gene and protein levels. The results presented on S3 and S4 Figs. show that benfotiamine alone had no effects on *TNF-α*- and *IL-6* mRNA (S3A, B Fig.) or TNF-α and IL-6 release (S4B,C Fig.). As shown in Fig. 4, benfotiamine decreased LPS-induced *TNF-α*-mRNA (Fig. 4A) and TNF-α release (Fig. 4B). The same holds for IL-6, which was down-regulated at both mRNA (Fig. 4C) and protein levels (Fig. 4D). Although benfotiamine showed tendency to up regulate mRNA expression of anti-inflammatory cytokine IL-10, when compared to LPS group (Fig. 4E), no statistically significant difference was observed. However, benfotiamine at 250 μM concentration induced significant stimulation of IL-10 release (Fig. 4F). Taken together, these data indicate that benfotiamine exerts anti-inflammatory properties by suppressing LPS-induced production of proinflammatory TNF-α and IL-6 and by stimulating the release of anti-inflammatory IL-10.

### Benfotiamine alters LPS-induced activation of MAPK and Akt signaling pathways

**Fig 5:**
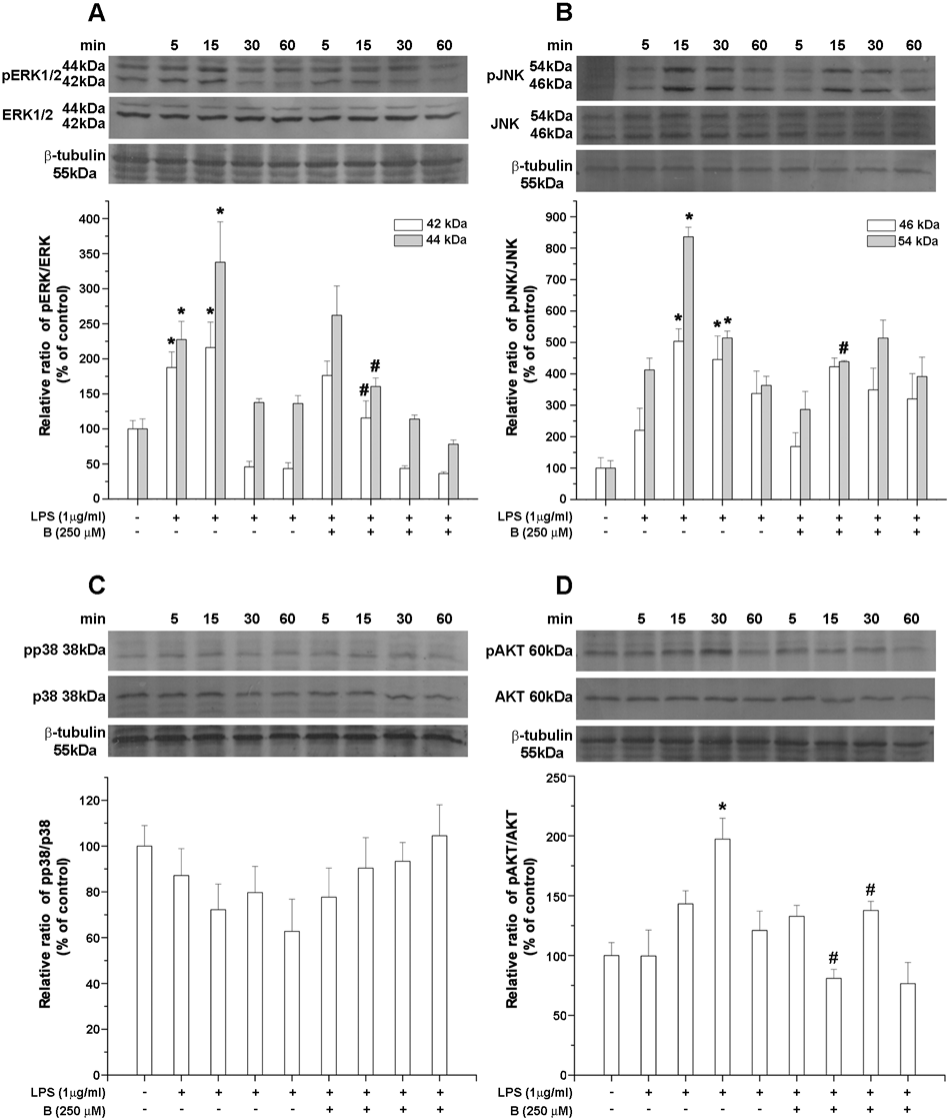
Quantitative Western blot analysis showing the effects of benfotiamine on MAP kinase signaling pathway. Expression levels of (**A**) pERK/ERK, (**B**) pJNK/JNK, (**C**) p38 and (**D**) pAKT/AKT were assessed 5–60 min following LPS stimulation. Bars represent mean expression ratio relative to β-tubulin ± SEM from *n* = 4 separate determinations. Significance levels shown inside the graphs: * - *p* < 0.05 control *vs*. LPS-induced BV-2 cells; # - LPS *vs*. benfotiamine pretreated LPS activated BV-2 cells.

A number of studies have demonstrated that MAPKs have important roles in modulating the expression of pro-inflammatory cytokines and iNOS in LPS-stimulated microglia. To analyze the molecular mechanism underlying the observed effects of benfotiamine, we further examined their inhibitory effect on phosphorylation of MAPK and Akt signaling pathways (Fig. 5), which are upstream signaling molecules in inflammatory responses. The cells were pre-treated with 250 μM benfotiamine for 30 min and then incubated with LPS (1 μg/ml) for 5–60 min. Treatment of the cells with LPS for different time periods was conducted to assess the capacity of benfotiamine to prevent different degrees of microglial activation. Treatment with LPS induced rapid phosphorylation of both 42-kDa and 44-kDa subunits of ERK/MAPK signaling pathway following a 5 and 15 min stimulation with LPS (Fig. 5A). While the peak ERK/MAPK expression was reached after 15 min LPS stimulation, the values leveled off to a control like amount after 30 and 60 min activation times. Notably, pretreatment with benfotiamine significantly reduced the level of phosphorylation of both ERK subunits for the 15 minutes LPS stimulation.

Treatment with LPS transiently activated JNK signaling pathway by inducing the phosphorylation of 46-kDa and 54-kDa subunits that peaked for the 15 min and remained elevated for the 30 min stimulation (Fig. 5B). In cells pre-treated with benfotiamine, on the other hand, phosphorylation of 54-kDa was effectively inhibited for the 15 min LPS stimulation. The p38 signaling pathway was not affected by LPS (Fig. 5C). Hence, additional preincubation with benfotiamine also had no influence on the pp38/p38 level. Treatment with LPS elevated the pAkt/Akt level after 30 min stimulation. This effect was effectively counteracted through preincubation with benfotiamine (Fig. 5D). Together, these data suggest that benfotiamine potently inhibits the peak changes in the protein levels of pERK, pJNK and pAkt caused by the LPS activation.

### Benfotiamine alleviates LPS-induced NF-κB translocation to nucleus

**Fig 6:**
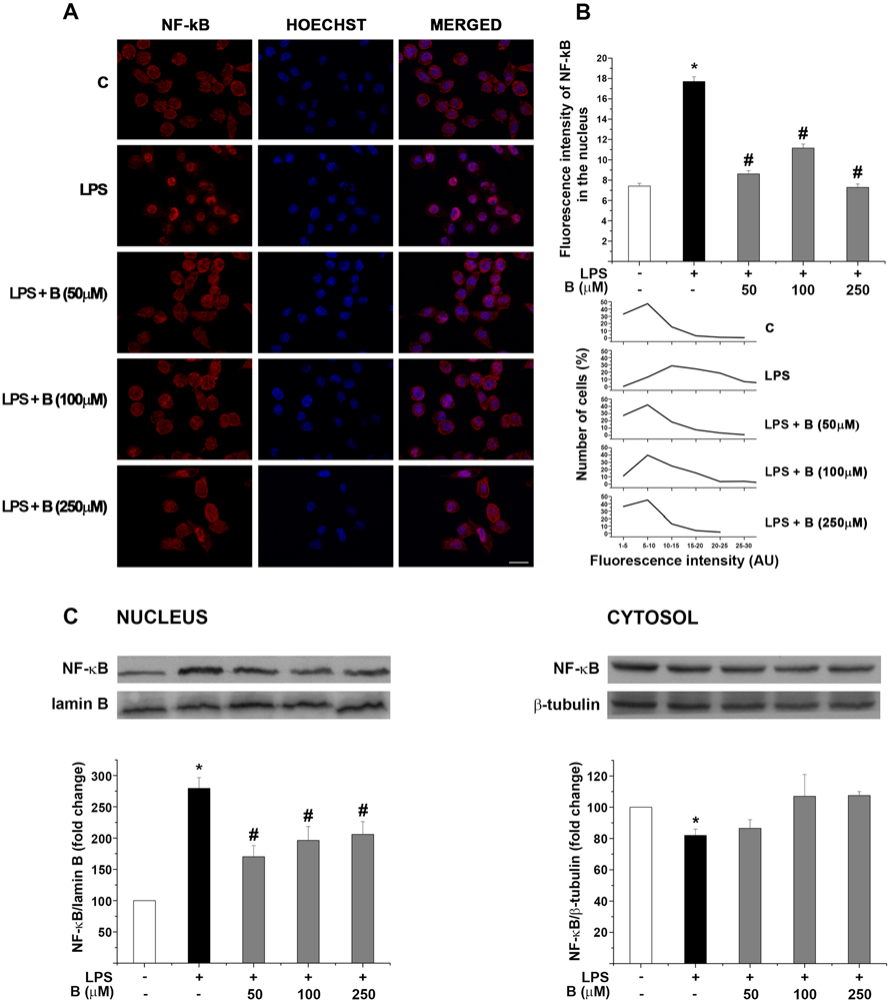
Effect of benfotiamine on LPS—induced nuclear translocation of NF-κB/p65. (**A**) Nuclear translocation of p65/NF-κB subunit was assessed by immunofluorescence labeling against p65 (red) and Hoechst nuclear fluorescence labeling (blue). (**B**) Nuclear fluorescence intensity of p65 was measured in > 200 hundred cells per experimental group, using ImageJ software and the results were presented in arbitrary units (lower graph). Data were binned (5 AU steps) according to fluorescence intensity and were represented as mean cumulative percentage ± SEM (upper graph). (**C**) Effect of benfotiamine on LPS—induced translocation of p65 from cytosolic to nuclear compartment was confirmed by Western blotting. Relative p65/β-tubulin abundance is expressed relative to the same abundance in control culture (100%) ± SEM from *n* = 4 separate determinations. Significance levels shown inside the graphs: * - *p* < 0.05 control *vs*. LPS-induced BV-2 cells; # - LPS *vs*. benfotiamine pretreated LPS activated BV-2 cells. Scale bar: 20 μm.

To determine whether the effects of benfotiamine in BV-2 cells were mediated *via* NF-κB signaling pathway, we analyzed nuclear translocation of NF-κB/p65 subunit, which is a critical step for the activation of this signaling pathway. BV-2 cells were pre-treated with benfotiamine (50, 100 and 250 μM) for 30 min and then treated with LPS (1 μg/ml) for 30 minutes. Treatment with benfotiamine alone did not alter nuclear p65 fluorescence intensity in all investigated dosages (S5A, B Fig.). By contrast, treatment with LPS induced a remarkable increase in nuclear the NF-κB/p65, as evidenced by a significant increase in nuclear p65 fluorescence intensity (Fig. 6A). Notably, the nuclear NF-κB/p65 protein level decreased significantly upon pre-treatment with benfotiamine in all concentrations tested. Mean nuclear NF-κB/p65 fluorescence intensities, collected from whole images are summarized in Fig. 6B. In BV-2 cells treated with benfotiamine, nuclear NF-κB/p65 intensities were comparable with the intensity in control cells, indicating that benfotiamine induced nuclear-to-cytoplasmic distribution of NF-κB/p65 similar to that in control cells. Distribution of relative nuclear NF-κB/p65 fluorescence intensity (arbitrary scale 1–30) in culture populations is presented in Fig. 6B (down). In control BV-2 cells, majority of cells (over 80%) showed nuclear NF-κB/p65 fluorescence intensity in the range of 1–10 AU, indicating poor nuclear p65 distribution. In cells treated with LPS over 90% exhibited the fluorescence intensity greater than 1–10 AU, with more than 30% of cell population exhibiting relative nuclear NF-κB/p65 intensity in the range of 20–30 AU. In cells pre-treated with benfotiamine at all tested concentrations, the distribution of relative nuclear NF-κB/p65 fluorescence was similar to control. Inhibition of NF-κB nuclear translocation by benfotiamine was additionally confirmed by p65 western blotting in nuclear extracts of BV-2 cells (Fig. 6C). These results together strongly suggest that benfotiamine alleviates LPS-induced NF-κB activation by preventing nuclear translocation of NF-κB/p65subunit.

Scale bar: 20 μm.

### Benfotiamine inhibits LPS-induced microglial activation through ERK, JNK and AKT pathways

**Fig 7:**
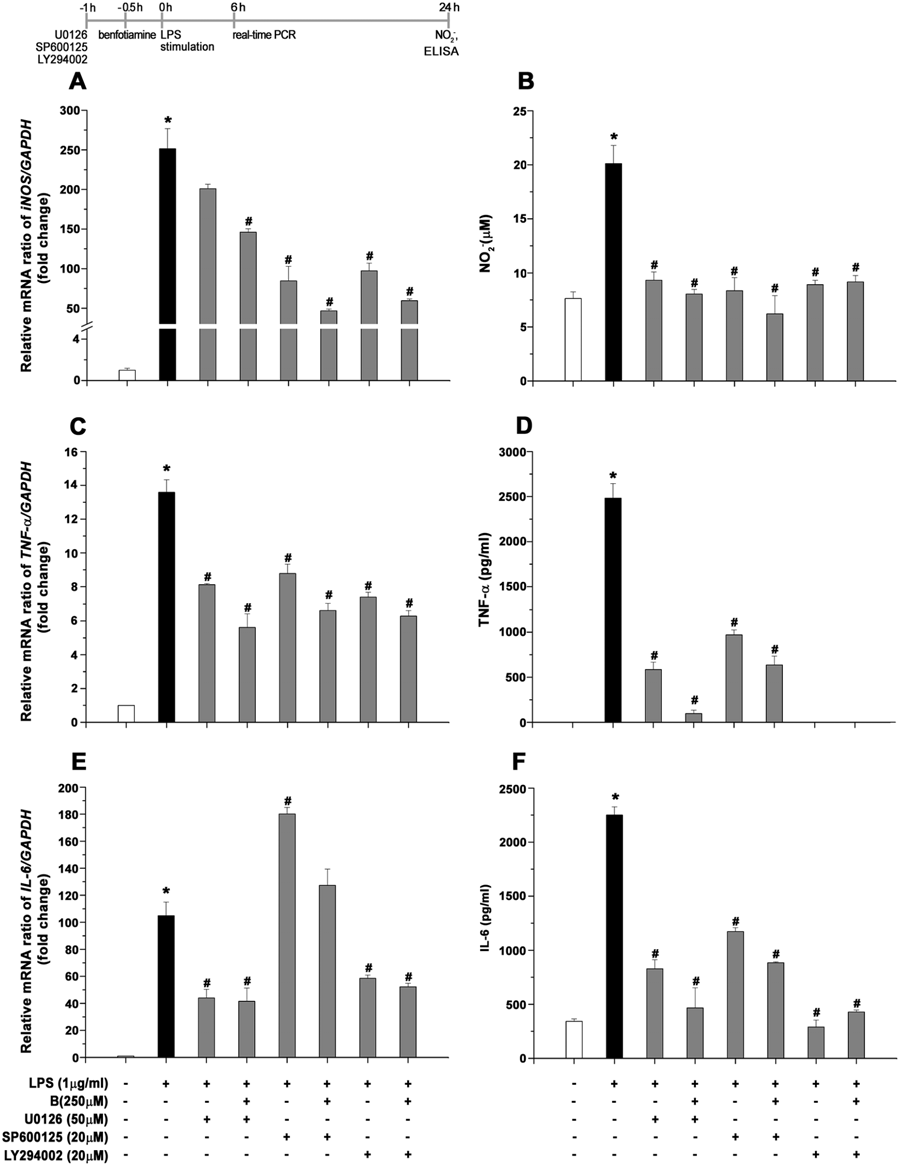
Effect of pharmacological inhibitors on iNOS, TNF and IL6 gene expression followed by NO, IL-6 and TNF-α production. (**A, C, E**) Expression of iNOS, TNF and IL6 at mRNA level in BV-2 cells. Expression of iNOS, TNF and IL6-mRNA was assessed by RT-PCR, in control culture (white bar), LPS-treated culture (black bar), cultures pre-treated with U0126 (50 μM), SP600125 (20 μM) or LY294002 (20 μM) in presence or absence of benfotiamine (gray bars), 6 h following addition of LPS. iNOS, TNF and IL6-mRNA abundance was expressed relative to the abundance of GAPDH-mRNA, as an internal control. (**B, D, F**) The cultured supernatants were collected and analyzed for NO using Griess method, or TNF-α and IL6 production with ELISA. The data represent the mean ± SEM (n = 3), *P<0.05 control vs. LPS-induced BV-2 cells, # LPS vs. benfotiamine pretreated LPS activated BV-2 cells.

To confirm the involvement of the ERK1/2, JNK and Akt signaling pathways in the anti-inflammatory effects of benfotiamine, we examined the effect of their pharmacological inhibitors on microglial activation. Using specific inhibitors for ERK1/2 (U0126), JNK (SP600125) and Akt (LY294002), we investigated LPS-induced mRNA levels of iNOS, TNF-α and IL-6, as well as NO, TNF-α and IL-6 production in BV-2 cells. BV-2 cells were pretreated with U0126, SP600125 and LY294002 for 30 minutes with subsequent incubation with benfotiamine (250 μM) for 30 minutes and stimulated with LPS. As shown in Fig. 7A, SP600125 and LY294002, like benfotiamine, significantly suppressed LPS-induced iNOS gene expression by 66 and 61%, respectively. In contrast, U0126 had no effect on mRNA iNOS expression while benfotiamine decreased iNOS gene expression. In addition, pretreatment with U0126, SP600125 and LY294002 significantly suppressed LPS-induced NO production by 54, 58 and 56%, respectively (Fig. 7B). Benfotiamine failed to show some additive effect. On the other hand, U0126, SP600125 and LY294002 reduced LPS-induced cytokine up-regulation. U0126 and LY294002 pretreatment resulted in a significant reduction of LPS-induced TNF-α (by 40 and 45%) and IL-6 (by 58 and 56%) mRNA expression (P < 0.05). In addition, subsequent incubation with benfotiamine also displayed significant reduction of TNF-α and IL-6 mRNA expression (Fig. 7 C, E). SP600125 reduced the elevation of TNF-α gene expression by 35,3% (P < 0.05), but resulted in increase in IL-6 gene expression. However, all three inhibitors in presence or absence of benfotiamine resulted in significant decrease of LPS—induced NO, TNF-α and IL-6 production (Fig. 7 B, D, F). Thus, these data collectively suggest that ERK1/2, JNK and AKT play a key role in the anti-inflammatory effects of benfotiamine.

## Discussion

Chronic and progressive neurodegeneration is generally associated with neuroinflammatory reaction mediated by resident glial cells in the brain — microglia and astrocytes. Hence, the control over the extent and duration of neuroinflammation through the modulation of glial response arose as a promising approach for treatment of neurodegenerative diseases. This was the rationale to explore the potency of benfotiamine to prevent inflammatory response in LPS activated BV2 microglial cells. The results of our study demonstrated that pretreatment with benfotiamine prevents the morphological changes evoked by LPS activation, decreases the production of NO, expression iNOS, COX-2, Hsp70 and modulates the release of master cytokines TNF-α and IL-6 by interfering with ERK1/2, JNK and NF-κB signaling pathways.

Reactive phenotypes in cultured microglia can be evoked by diverse inflammatory challenges, such as LPS-induced toxicity [38,39,40]. Once activated in an inflammatory environment, microglia acquires the macrophage-like capabilities, including amoeboid cell shape, migration, production of inflammatory cytokines and phagocytosis. One of the important markers of microglial morphology is the organization of F-actin fibers [41,42,7]. Our data showed that benfotiamine induced prominent alterations in the morphology of LPS-activated BV-2 cells, by a mechanism engaging: (i) the reorganization of the actin cytoskeleton, (ii) reduction of dense fasciation of membrane-bound stress fibers and (iii) promoting the stress fibers relocalization throughout the cell. The LPS-activated BV-2 cells exhibited dense network of F-actin fibers forming numerous membrane ruffling’s at the cell border, whereas pretreatment with benfotiamine transformed the cells to be small and ovoid in shape, with smooth cell edges. Benfotiamine putatively exerts its protective effects against microglial activation by suppressing the formation of membrane ruffling’s which are found at the front edge of activated microglia and represent the driving force in chemotaxis [43]. In fact, benfotiamine treated LPS-induced BV-2 cells retained the shape that is characteristic of non-stimulated microglia. Concomitant with morphological changes, biochemical alternation occurred as well.

Another hallmark of activated microglia is the production of pro-inflammatory mediators and cytokines, which trigger an inflammatory cascade and perpetuate inflammatory processes associated with several neurodegenerative diseases. Our data is consistent with benfotiamine-induced decrease of NO production and expression of proinflammatory cytokines TNF-α and IL-6 by LPS-activated BV-2 cells.

NO is an important signaling molecule with diverse regulatory roles in the nervous system [44,45]. It is generated endogenously by catalytic action of iNOS. High levels of NO induce COX-2 expression, additional effector molecule implicated in inflammatory neuropathology. COX-2 is an enzyme encoded by the PTGS2 gene and its activation is associated with various inflammatory diseases [46]. Therefore, a compound capable of downregulating COX-2 could potentially possess anti-inflammatory activities. It has been shown that benfotiamine reduces production of NO and inhibits iNOS protein expression in LPS-stimulated macrophages [34]. Consistent with previous study, we reported that pretreatment with benfotiamine inhibited NO secretion and suppressed iNOS and COX-2 at both the gene and protein levels in LPS-stimulated BV-2 cells. In addition, benfotiamine reduced expression and release of TNF-α and IL-6, which are the cytotoxic mediators linked with the development of chronic inflammatory and autoimmune diseases [47]. Specifically, TNF-α signaling recruits different signaling mediators including caspases, NF-κB and MAPK, eventually leading to transcriptional activation of inflammatory genes [48,49]. The IL-6 modulates phagocytic activity and induces morphological alterations in microglia [50]. On the other hand, IL-10 inhibits the LPS-induced increase in IL-1β and TNF-α [51] and modulates PI3K pathway [52,53,54]. Taken together, we conclude that benfotiamine shifts BV-2 microglial cells from inflammatory toward more quiescent cell state, as it reduces iNOS, TNF-α and IL-6 gene and protein expression and slightly increases IL-10 production in response to LPS.

In microglial cells, NF-κB regulates a number of proinflammatory genes, including iNOS, PTGS [55], TNF-α and IL-6 [56,57,58]. We found that benfotiamine significantly downregulates the proinflammatory mediators and cytokines in LPS-activated BV-2 cells, through modulation of multiple signaling pathways. Namely, the importance of ERK1/2 in iNOS and COX-2 expression [59,45] or microglia activation, migration and production of cytokines, such as IL-6 is well established [60,61,62]. On the other hand, JNK signaling pathway is involved in morphological modification, cytokine transcription [63,64,65] and it was proposed to act as a co-mediator in activation of microglia [66,67,68]. Furthermore, the Akt/PKB signaling pathway seems to be required for the activation of inflammatory responses in microglial cells [69]. In this study, we were able to demonstrate that benfotiamine significantly reduced the LPS-induced increase in phosphorylated levels of ERK1/2, JNK and Akt/PKB. Further studies with pharmacological MAPK inhibitors revealed that JNK and Akt specific inhibitor SP600125 and LY294002 led to significant reduction of LPS-induced iNOS mRNA expression and NO production, whereas inhibition of ERK1/2 signaling by U0126 displayed no effect on iNOS mRNA, suggesting iNOS expression is induced mainly through JNK1/2 and Akt signaling. Indeed, suppression of iNOS induction and NO production in reactive microglia by JNK1/2 inhibitors has been consistently reported [67, 70]. Moreover, inhibition of Akt phosphorylation is found to be involved in inhibition of iNOS in microglia [71], while the role of ERK seems controversial, as both, inhibition or no effect by ERK1/2 inhibitors have been reported [67,72]. Benfotiamine in these experiments failed to show some additive effect. In regard to expression of proinflammatory cytokines, inhibition of ERK1/2, JNK and Akt resulted in a reduction of the LPS-stimulated TNF-α and IL-6 release, demonstrating that benfotiamine suppresses LPS-induced cytokine production collectively via these signaling pathways without exerting any additional effect on activated microglia. Several studies have demonstrated that the PI3K/Akt pathway is the prerequisite for the activation of NF-κB leading to elevation of proinflammatory mediators in BV2 cells [73, 69]. It is known that activation of NF-κB signaling cascade requires translocation of NF-κB/p65. Benfotiamine potency to inhibit NF-κB activation was previously shown in an in vivo model of diabetes [22], as well as in vitro, in LPS-activated macrophages [34]. Our data demonstrated that benfotiamine reduced the LPS-stimulated intranuclear accumulation of NF-κB/p65 and decreased a fraction of cells with activated NF-κB signaling cascade. Thus, based on these results, we suggest that benfotiamine inhibits translocation of NF-κB/p65 into the nucleus and consequently alleviate the transcription of proinflammatory genes.

In conclusion, the present observations identify a potential anti-inflammatory role of benfotiamine in LPS-activated microglia, mainly through the inhibition of ERK1/2, JNK and Akt activation, by interference with NFkB activity. Moreover, our results opens the possibility that benfotiamine might be useful in treatment of pathologies that involve chronic inflammation, observed in some neurodegenerative diseases, such as Alzheimer’s, Parkinson’s disease or multiple sclerosis. Although, the neuroprotective actions of benfotiamine need to be explored further, these findings suggest that additional in vivo studies will provide a feasible strategy to modulate an inflammatory response in the CNS.

## Supporting Information

S1 Fig Functional characterization of benfotiamine effects in BV-2. (A) Dynamic monitoring of BV-2 cell activation was analyzed using xCELLigence RTCA analyzer. Real-time impedance measurement demonstrated slow, gradual increase in cell impedance in all examined groups. (B) The effect of benfotiamine on morphological changes. The cell morphology was observed with phase-contrast microscopy, followed by Phalloidin /Hoechst staining (red/blue). (C) The quantification of cell size was performed using Axiovision 4.6 software (n = 3). (D) Cell viability was evaluated by crystal violet assay. Each value indicates the mean ± SEM (n = 3).Scale bar: 20 μm.
(TIF)

S2 Fig Benfotiamine does not affect cell viability of BV2 microglial cells assessed by trypan blue exclusion test. The cells were pretreated with benfotiamine at indicated dosages for 30 minutes in presence or absence of LPS for additional 24h. Data are represented as mean ± S.E.M. of five independent experiments performed in triplicate.
(TIF)

S3 Fig The cells were pretreated with benfotiamine at indicated dosages for 30 minutes and incubated for additional 6h to analyze mRNA levels by means of real-time PCR. The gene expression was normalized to the endogenous control GAPDH. Data are represented as mean ± S.E.M. of three independent experiments.
(TIF)

S4 Fig Effect of benfotiamine on NO, TNF-α and IL6 production in control BV2 cells. BV2 cells were pretreated with benfotiamine for 30 minutes and incubated for additional 24h in absence of LPS. NO production was assessed by Griess assay. The amounts of TNF-α and IL6 in cell culture supernatants were obtained using ELISA. Data are represented as mean ± S.E.M. of three independent experiments.
(TIF)

S5 Fig Effect of benfotiamine on NF-κB/p65 activity. (A) Immunofluorescence images of cells stained with antibody against p65 subunit of NF-κB (red) and Hoechst (blue). (B) Quantification of fluorescence intensity of NF-κB/p65 in the nucleus, evaluated with ImageJ software (B, top). Mean values of fluorescence intensity ± SEM, expressed in arbitrary units (B, down). Distribution of fluorescence intensity in groups treated with benfotiamine is similar to control group. The data represent the mean±SEM (n = 4). Scale bar: 20 μm.
(TIF)

S6 Fig Benfotiamine suppressed LPS-induced expression of Hsp70 in BV2 cells. The cells were pre-treated with the indicated concentration of benfotiamine for 30 minutes, followed by treatment of LPS (1 μg/mL) for 24 h. A) shows representative image of the western blot B) show the optical densities of Hsp70 normalized to the loading control β-actin (n = 4). Stimulation of BV-2 cells with LPS leads to a strong increase in the production of Hsp70, while benfotiamine (250 μM) treatment decrease the expression of Hsp70. *P<0.05 control vs. LPS-induced BV-2 cells, # LPS vs. benfotiamine pretreated LPS activated BV-2 cells.
(TIF)
