# Supplementary figures and images for: Benfotiamine Attenuates Inflammatory Response in LPS Stimulated BV-2 Microglia
Source: PLoS One. 2015 Feb 19;10(2):e0118372. doi: 10.1371/journal.pone.0118372 (PMC4335016; doi:10.1371/journal.pone.0118372)

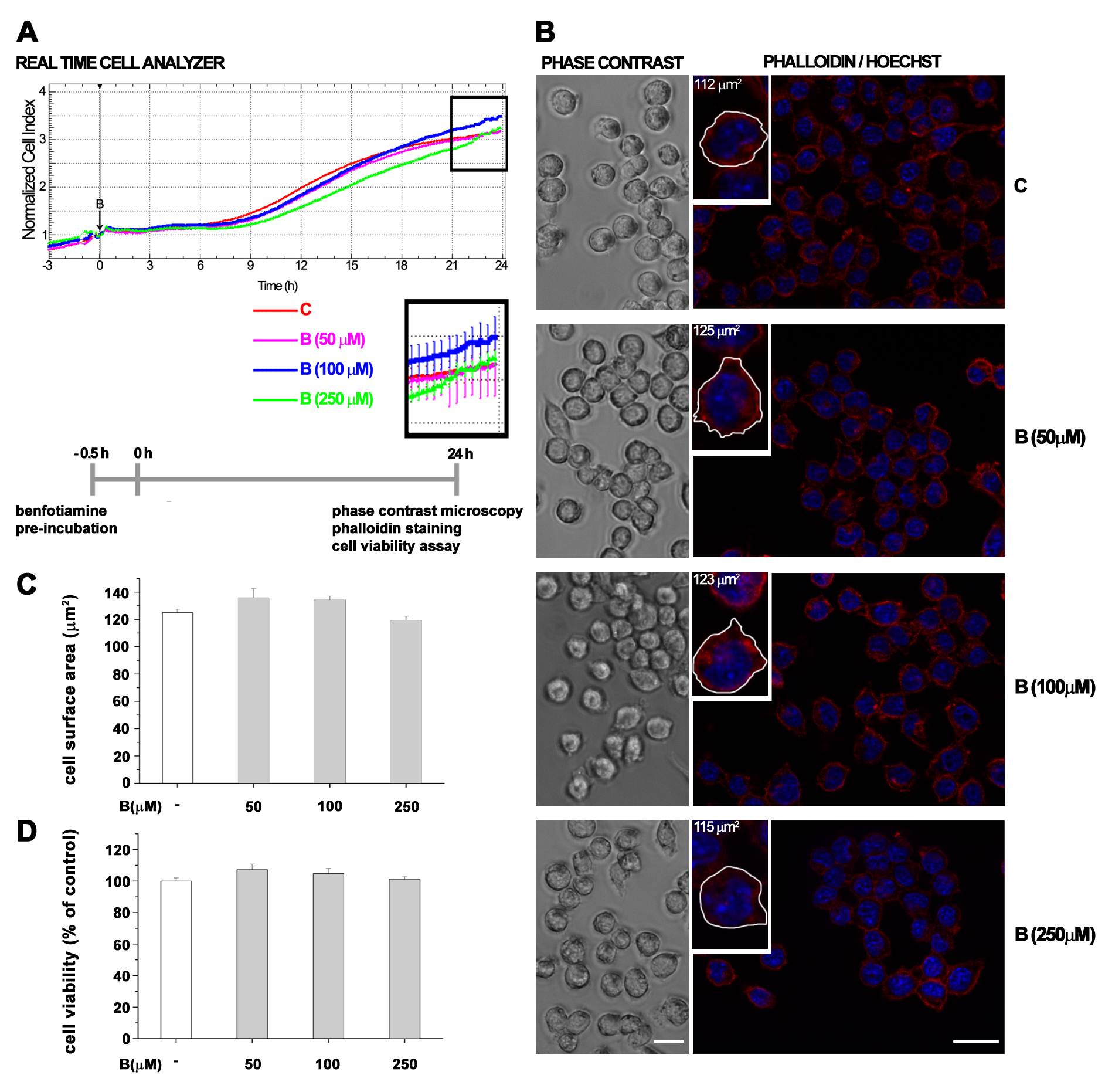

Supplement: S1 Fig — (A) Dynamic monitoring of BV-2 cell activation was analyzed using xCELLigence RTCA analyzer. Real-time impedance measurement demonstrated slow, gradual increase in cell impedance in all examined groups. (B) The effect of benfotiamine on morphological changes. The cell morphology was observed with phase-contrast microscopy, followed by Phalloidin /Hoechst staining (red/blue). (C) The quantification of cell size was performed using Axiovision 4.6 software (n = 3). (D) Cell viability was evaluated by crystal violet assay. Each value indicates the mean ± SEM (n = 3).Scale bar: 20 μm. (TIF) [file pone.0118372.s001.tif]

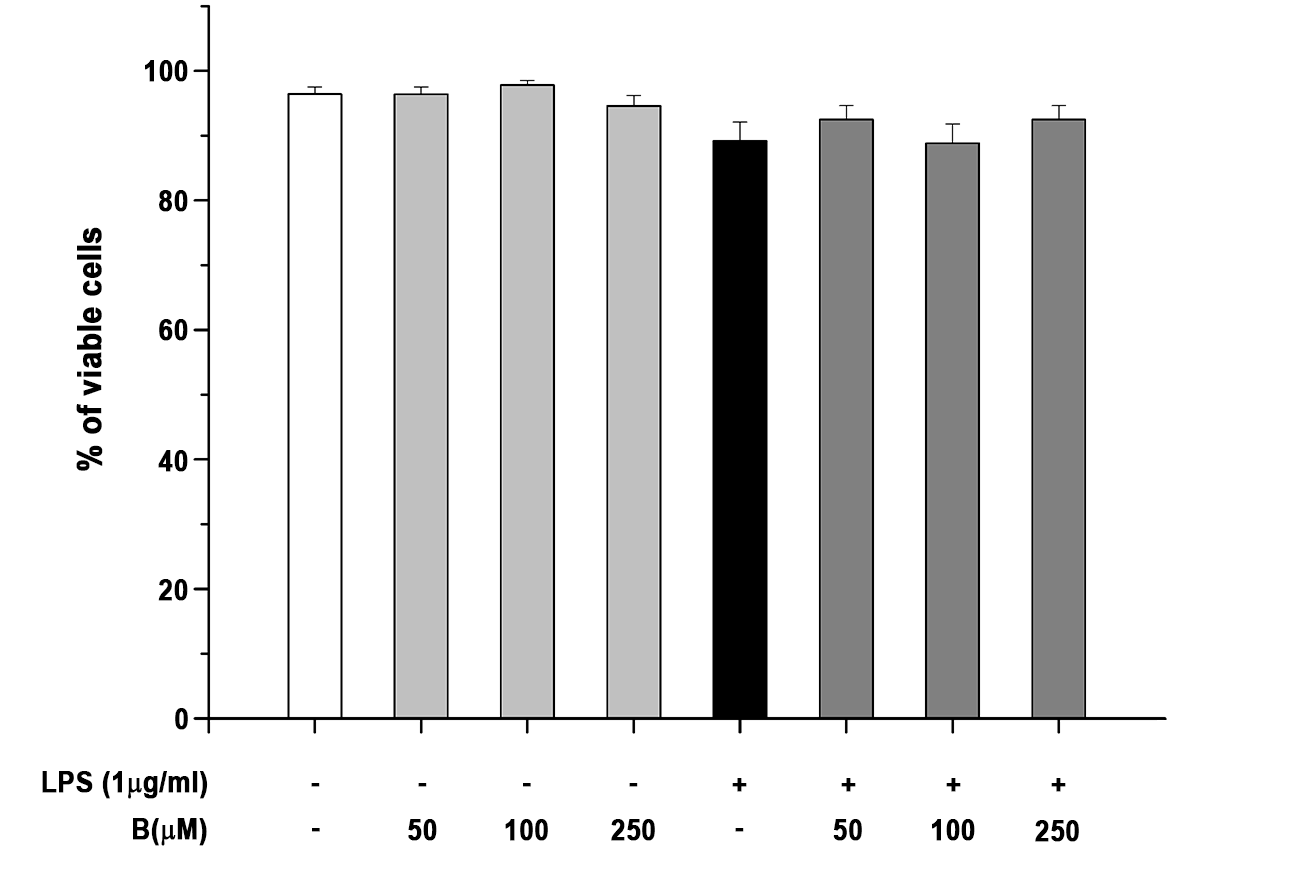

Supplement: S2 Fig — The cells were pretreated with benfotiamine at indicated dosages for 30 minutes in presence or absence of LPS for additional 24h. Data are represented as mean ± S.E.M. of five independent experiments performed in triplicate. (TIF) [file pone.0118372.s002.tif]

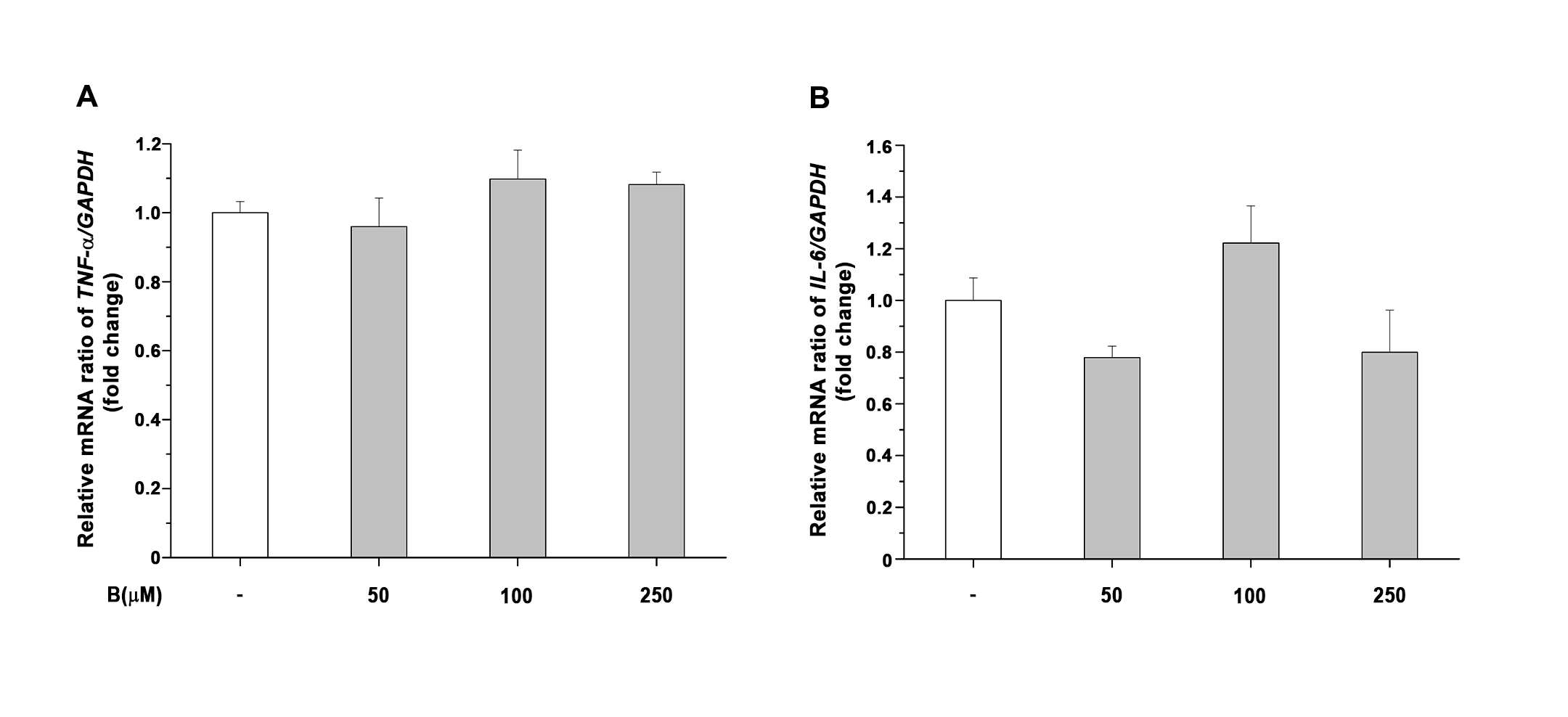

Supplement: S3 Fig — The gene expression was normalized to the endogenous control GAPDH. Data are represented as mean ± S.E.M. of three independent experiments. (TIF) [file pone.0118372.s003.tif]

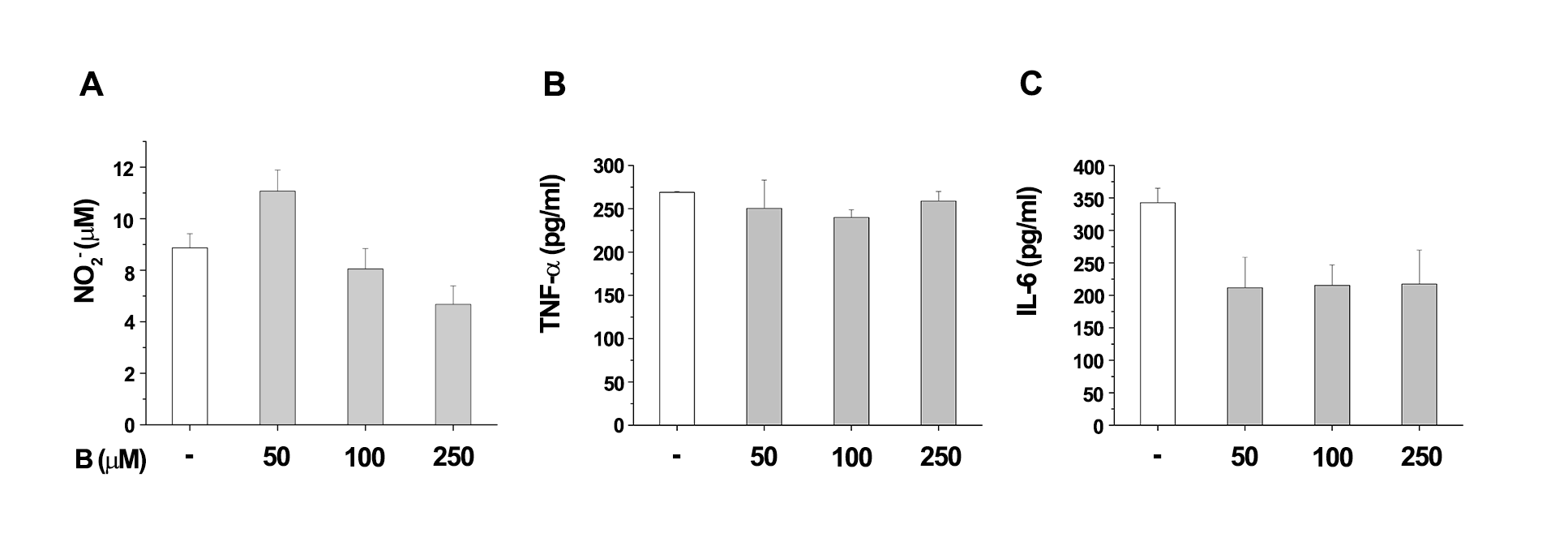

Supplement: S4 Fig — BV2 cells were pretreated with benfotiamine for 30 minutes and incubated for additional 24h in absence of LPS. NO production was assessed by Griess assay. The amounts of TNF-α and IL6 in cell culture supernatants were obtained using ELISA. Data are represented as mean ± S.E.M. of three independent experiments. (TIF) [file pone.0118372.s004.tif]

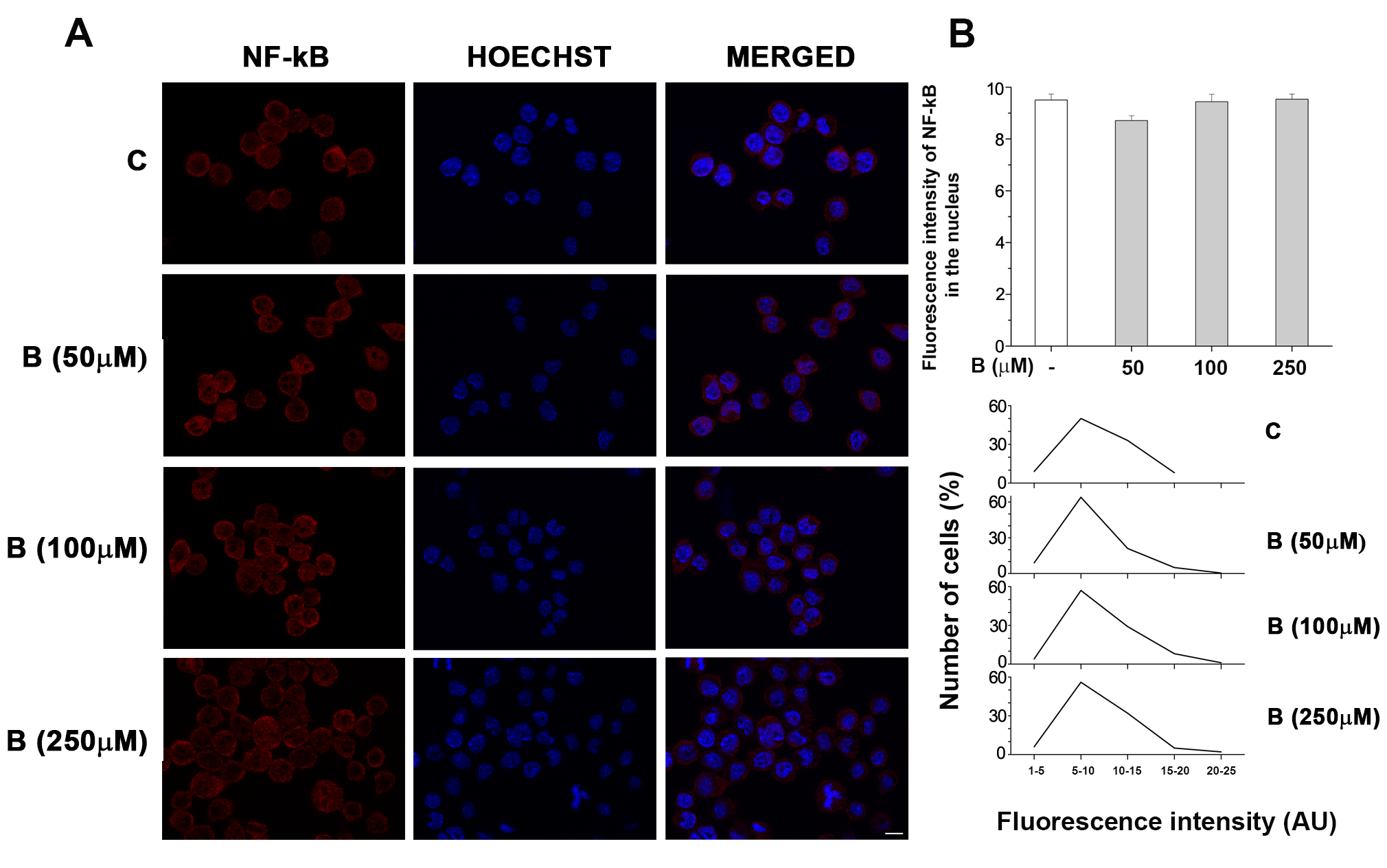

Supplement: S5 Fig — (A) Immunofluorescence images of cells stained with antibody against p65 subunit of NF-κB (red) and Hoechst (blue). (B) Quantification of fluorescence intensity of NF-κB/p65 in the nucleus, evaluated with ImageJ software (B, top). Mean values of fluorescence intensity ± SEM, expressed in arbitrary units (B, down). Distribution of fluorescence intensity in groups treated with benfotiamine is similar to control group. The data represent the mean±SEM (n = 4). Scale bar: 20 μm. (TIF) [file pone.0118372.s005.tif]

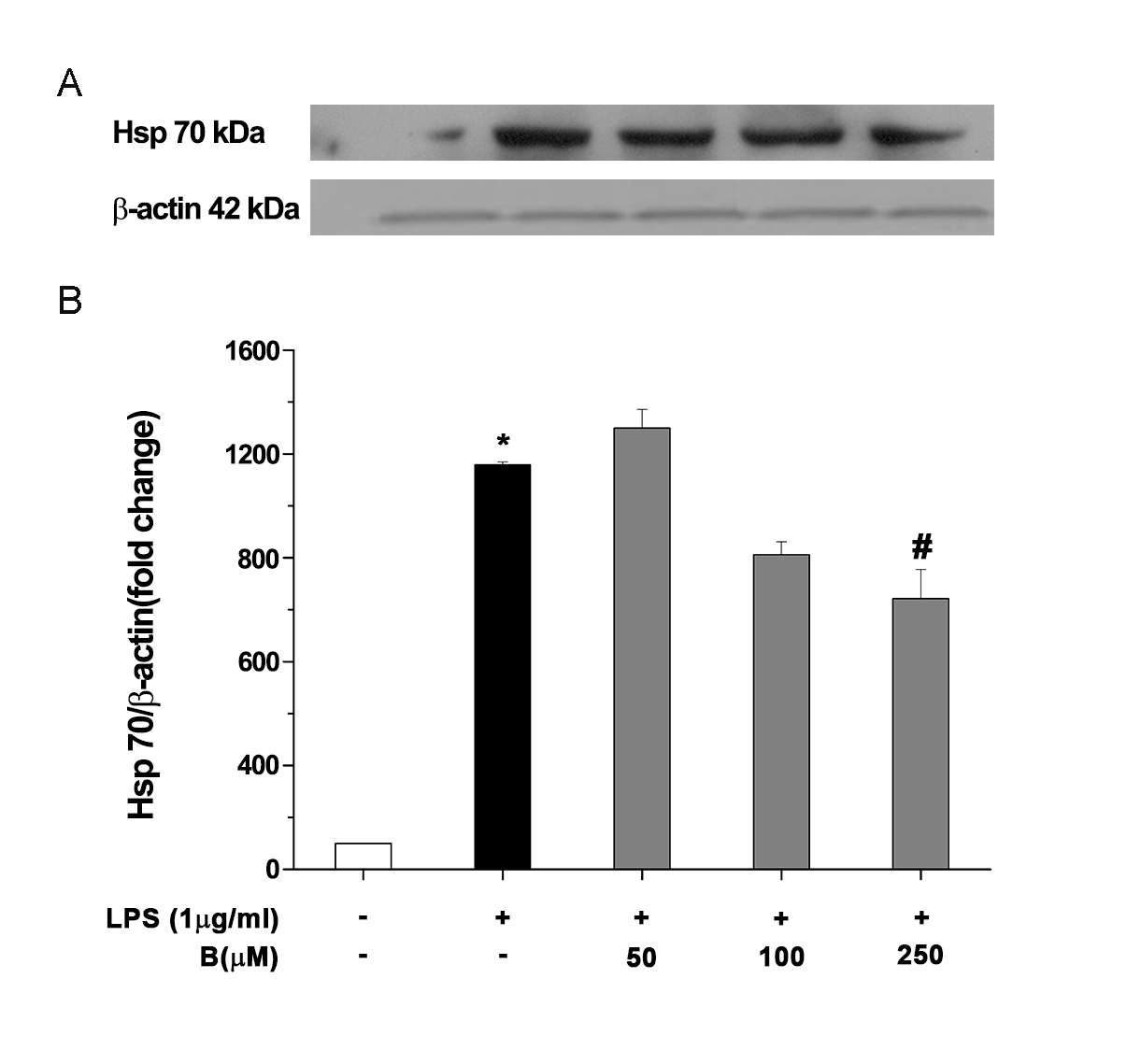

Supplement: S6 Fig — The cells were pre-treated with the indicated concentration of benfotiamine for 30 minutes, followed by treatment of LPS (1 μg/mL) for 24 h. A) shows representative image of the western blot B) show the optical densities of Hsp70 normalized to the loading control β-actin (n = 4). Stimulation of BV-2 cells with LPS leads to a strong increase in the production of Hsp70, while benfotiamine (250 μM) treatment decrease the expression of Hsp70. *P<0.05 control vs. LPS-induced BV-2 cells, # LPS vs. benfotiamine pretreated LPS activated BV-2 cells. (TIF) [file pone.0118372.s006.tif]
